# A Physiological Essay on the Thymus Gland

**Published:** 1845-07

**Authors:** 


### Art. XV.

*A Physiological Essay on the Thymus Gland.* By John Simon, f.r.s., Demonstrator of Anatomy in King's College, London; &c. &c.—*London,* 1845. 4to, pp. 100. With 53 Wood-Engravings.

This elaborate and beautifully-illustrated Memoir is the first-fruit of the liberal arrangement made in Sir Astley Cooper's will, by which a sum of £300 was to be given as a triennial prize for the encouragement of original investigations in Physiology and Surgery. The judges appointed to fix the subjects for investigation, and to decide upon the merits of the competing essays, not unnaturally or injudiciously selected, on the first occasion, a topic which had occupied the attention of the munificent founder of the prize; and about a year since they made their award in favour of the present Essay. During this interval the author has carefully reviewed his work, and has made various improvements in it,—having not only verified his former observations, but having also, in some instances, obtained additional evidence on points where he formerly had doubts.

We do not hesitate to affirm, that no more complete description is extant of any organ in the animal body, than that which Mr. Simon has here furnished in regard to this obscure and ill-understood part of the organism. He has not only carefully analysed its structure by the microscope, in animals in which it attains its highest development, but he has traced it through an extensive series of animals, including typical forms of most of the principal groups in which it occurs; he has also scrutinized its embryonic development, from the earliest period at which it is discoverable; and he has examined the structure of the spleen, thyroid body, and supra-renal glands, for the sake of comparison with it. We must content ourselves with briefly stating the conclusions at which he has arrived; these are not merely interesting in themselves, as affording a plausible solution of a *quæstio vexata,* which has much troubled anatomists and physiologists, but they are of much importance, from their bearing on the doctrines of general physiology.

We must pass over, for want of space, the historical introduction, principally derived from the learned work of Dr. Haugsted (Copenhagen, 1832), in which a summary has been given of all that has been previously written, both as to the structure of this body and its supposed uses. The history is not uninstructive, however, as showing the futility of theorizing, as to the functions of this body, upon such imperfect knowledge of its structure and relations as our forefathers possessed. Sir Astley Cooper's work is deservedly praised, as having "finally fixed the correct view of the structure of the gland as completely as could be done without the use of the microscope." But the recent *perfectionnement* of that instrument, and the introduction of new views in regard to the constitution and actions of glands, founded on its revelations, have opened a novel field of research, which had been scarcely upturned in this quarter, previously to Mr. Simon's investigations.

The embryonic development of the thymus gland takes place nearly upon the same plan with that of glands in general. "By the naked eye, or with the assistance of a simple lens, its existence may be distinctly ascertained in fœtuses of about an inch and a half in length; and by careful manipulation it may be followed under the microscope in its earlier stages, even in embryos little more than half an inch long." The earliest form which Mr. Simon has been able to discover is that of a simple tube, closed at *both* ends; the walls of which consist of a very delicate homogeneous membrane; whilst its cavity contains granular matter. From certain appearances presented by this tube, he considers it probable that it is formed by the coalescence of a linear series of cells. Even at this period, therefore, the essential analogy and the essential difference between the thymus and other glands are manifest; for the latter, however complex they subsequently become, are first to be traced as simple tubes or follicles; but these are closed at *one* end only, and open at the other on some mucous surface. In the subsequent development of the thymus, the regular type of glandular production is followed; for it essentially consists in *the lateral growth of branching diverticula from the central tubular axis*. In the mature thymus, the primary tube, although it has increased pari passu with the growth of the region to which it belongs, is yet so hidden and eclipsed by the disproportionate bulk of its follicular extensions, that many anatomists have overlooked its existence or real nature, and have described the whole body as consisting of an aggregation of independent vesicles. Hence the result at which Mr. Simon has arrived, by the study of the embryonic development of the thymus, corresponds with that which Sir A. Cooper had obtained by the artificial distension of its cavities; but it corrects the latter in one particular—viz., as to the proportional size of the central cavity, or "reservoir" of Sir A. Cooper, which is not so great as was represented by him to be; the appearance of a large cavity having been artificially produced by the means employed for expanding and exhibiting the interior structure of the body.

The investigation as to the period of greatest development and activity of the thymus is one of much physiological importance. The usual idea on this point has been, that its greatest bulk is attained during the latter part of embryonic life; and that it must consequently exercise its chief functional activity during the intra-uterine period of existence, and have reference to the peculiarities of fetal life. The researches of Dr. Haugsted, however, afford most convincing proof that this is an error; since not merely the *absolute*, but the *relative* size of the gland undergo a great increase after birth. Thus, whilst the weight of a dog's thymus at birth may range up to *ten* grains, we find it subsequently increasing with such rapid paces, that after five months it weighs nearly *four hundred;*—a proportional increase of *forty* times, whilst the weight of the entire animal has increased only *twelve* or *sixteen* times. The data collected by Mr. Simon on this point fully confirm those of Dr. Haugsted; and he is, in consequence, fully justified in the statement that "the thymus can with no more propriety be referred to the needs and uses of fœtal life, than the mammæ of the female can be considered subservient to the period of uterogestation." In the human subject it appears that, during the period immediately succeeding birth, the activity of the growth of the thymus is greater than that of the body in general; and its functional energy seems then at its greatest. This rapid state of growth, however, soon subsides into one of less activity, which merely serves to maintain the proportion so acquired; and its absolute increase usually ceases at about the age of two years. From that time, during a variable number of years, it remains stationary in point of size; but, if the individual be adequately nourished, it gradually assumes the structure of fat,—a curious change, which, as will presently appear, is still more remarkable in certain other animals. The duration of its decay, and the epoch of its entire vanishing, are so uncertain, that no general statement can be made regarding them. Its principal loss of substance appears usually to take place about the period of puberty. The size of the thymus at each period, in proportion to that of the entire body, seems to undergo considerable variation, in accordance with the general activity of the nutritive functions in the individual, and with the amount of waste occasioned by exercise of its muscular tissue. Thus it is stated by Mr. Simon, that where the weight of the whole body at birth exceeds the average, the weight of the thymus exceeds the average in a disproportionate degree; whilst, if it be beneath the average, the thymus is disproportionately small. And it was long ago observed by Wharton, that if a young ox be put to the plough, the disappearance of its thymus is much accelerated. Mr. Simon is even disposed to believe that a great variation in its bulk may take place in the same individual within a few days, if not hours.

From the foregoing facts it appears that the thymus is to be regarded as an organ characteristic of the period of *growth*, or of that in which there is an excess of nutrition over waste. But it is no more possible to fix by a general rule, for all species, and for all individuals, the exact duration of its existence, or the date of its chief development, than it is to express in numerical terms the precise extent of that period of growth to which its usefulness applies. In proportion as the muscular powers of the system become vigorous, and the activity of the merely nutrient operations diminishes, in that proportion do we find the thymus disappearing. This is apparent, not only from the comparison of different individuals of the same species, but also from the comparison of the classes of birds and reptiles; for as the former are distinguished by the *lowest* and the latter by the *highest* activity of respiration and muscular movement—so do we find in the former the longest persistence of the thymus, and in the latter its quickest departure.

We have now to speak of the minute stucture of the gland in its mature state; and of the nature of the fluid found within it. The terminal vesicles, all of them communicating with the central tube or axis, are composed of a delicate homogeneous membrane, the basement-membrane of Mr. Bowman; whilst they are surrounded by a close capillary network, which lies in contact with the outer surface of the membrane, and is adapted to its various inflexions. The cavities of the vesicles contain a fluid, in which, as Hewson discovered, an immense number of microscopical corpuscles float. These corpuscles, which are about the size of those of the blood, are generally of discoid form, and seem rather to deserve the character of nuclei or cytoblasts, than that of cells. In specimens taken from animals past that period of life when the thymus is most active, Mr. Simon states that he has found cells, to which these dotted corpuscles presented the relation of nuclei; these cells are at first little larger than the corpuscles themselves, and contain a perfectly pellucid material; but as they grow, their contents become molecular, and they develop themselves into perfect fat-cells, which lie in the cavities of the gland, and in some instances completely fill them. The presence of fat-particles in the ordinary corpuscles appears to be indicated by the existence of a variable number of minute markings in each, giving to them a dotted appearance; these markings may present themselves as dark spots, to the number of two, three, four, or even five; or there may be only a single one, which is then found proportionably large, and possesses a high refracting power, like that which oily particles elsewhere exhibit; chemical analyses of the gland at the period of greatest activity, however, give but very slight traces of fat; both proximate and ultimate analysis accord in giving to its substance and contents a composition nearly allied to that of blood and muscle. This fact is of considerable importance as demonstrating the incorrectness of the theory of Tiedemann, Arnold, and others, that the office of the thymus is to separate, during intra-uterine life, a carbonaceous product from the blood, and thus to take the place of the respiratory organs in depurating it. The actual and ultimate nature of the secretion of the thymus, (so far as it can be ascertained,) is expressed by the formula for proteine; or, in other words, is nutrient matter.

The fourth chapter contains an elaborate account of the "Comparative anatomy of the thymus gland;" which Mr. Simon has ascertained to exist, not merely in mammalia, to which it was formerly supposed to be restricted, but also in birds and reptiles. Below the last-named class, however, he can discover no traces of it. It is in the hybernating species of the order Rodentia, that we find the greatest peculiarity in regard to this body. Instead of disappearing as the animal advances towards adult age, it seems to continue to increase; being found in the marmot not only to surround the base of the heart, but also to extend itself into the axilla and posterior mediastinum. But in this, as in other cases in which the thymus becomes a permanent organ, it does so under an altered character; namely, by a singular and striking transformation of its ultimate elements,—"*by developing its natural cytoblasts and fluid contents into a system of nucleated fat-cells, held within a limitary membrane.*" In the animals which hybernate less completely, these voluminous organs are not so fully developed; and in those which pass the winter in a state of activity, they are absent,—the thymus being merely a "temporary organ, as in other mammalia."

The existence of a thymus in birds was first asserted by Meckel; but the organ described by him under this name is stated by Mr. Simon to have none of the characters of the true thymus, and to consist of nothing else, even in the youngest specimens, but ordinary adipose tissue. But having detected this fallacy, he has also adduced satisfactory evidence of the real existence of a thymus in birds, although it never reaches a high grade of development, and presents, in fact, a form quite rudimentary, —that of a semi-transparent ampullated tube, following the line of the superficial cervical vessels, and containing the characteristic dotted corpuscles of this organ in the mammalia. Its development appears to stop, and its functions to cease, at a very early period. The physiology of the thymus is said by Mr. Simon to be better illustrated in the class of reptiles than in any other; yet there has been the greatest misapprehension on the subject. The real thymus has been described as a thyroid gland, and *vice versa;* and Becker and Haugsted, who profess to have thoroughly examined the parts referred to, agree in positively denying the existence of a true thymus in reptiles. According to Mr. Simon, "the careful employment of the microscope in practised hands, for investigations of this nature, precludes the possibility of error. The thymus and thyroid can be no more confounded with each other, or with fat, than the liver and kidney can be mistaken for each other or for muscle. The tubulo-vesicular structure of the thymus, the vesicles of the thyroid, the distinct limitary membrane and peculiar contained corpuscles of each, will serve to identify and distinguish these organs with *absolute certainty.*" Generally speaking, the thymus of reptiles is large and persistent, and undergoes a metamorphosis into fatty tissue, as in the hybernating mammalia. The general rule of its connexion with pulmonic respiration holds good among the Batrachian reptiles, as in fishes. During the tadpole state or first condition of the frog and its allies, no trace of thymus can be detected; but as soon as the pulmonic respiration is beginning to be established, the organ makes its appearance. In those of the Perennibranchiate Batrachia, whose respiration is rather branchial than pulmonic, Mr. Simon has been unable to detect it. In fishes, after repeated and careful search in about twenty genera, he has been unable to discover any signs of a thymus; and there is no reason to believe that any analogous organ exists in the invertebrata.

The chief results of his comparative examination are briefly stated by Mr. Simon, as follows:

"1. *The presence of the gland* is coextensive with pulmonary respiration. 2. Its *shape and position* are variable and unimportant. 3. Its *size and duration* are, generally speaking, in proportion to the habitual or periodical inactivity of the animal. 4. Where it remains as a persistent organ, it is usually but one of several means for the accumulation of nutritive material; its continuance, under such circumstances, is generally accompanied—though, in some instances, superseded —by a peculiar accessory contrivance, the fat-body." (p. 64.)

In the fifth chapter, the morphology of the thymus is considered; its structure being compared to that of the true glands; and some important considerations being offered, in regard to the nature of its elementary operations. As already mentioned, it has much in common with true glands, both in its minute composition, and in the disposition of its parts; but the corpuscles which its tubes and follicles contain are rather *cytoblasts* than perfect cells; and the cavity into which all the diverticula open is closed, instead of possessing an excretory duct. The following views in regard to the character and functions of the cytoblasts, although very different from those now generally entertained, have much plausibility, and merit an attentive examination.

"There are many reasons for believing that the so-called nucleus or cytoblast of a cell is its essential part, and capable of fulfilling by itself the entire functions which have been generally ascribed to the wall of the complete cell; and it is highly important for the physiological understanding of the glands without ducts, that the grounds of this belief should be examined.

"It appears that in the development of secretory cells, there are the following steps:—First, the formation of the nuclei;—Secondly, the deposition of material around them; which step seems the first evidence of their peculiar function;—Thirdly, the isolation of this material by the growth of a membrane around it,—in other words, the completion of a cell, which has now all its elements, nucleus, membrane, and contents;—Fourthly, a stage of apparent quiescence, during which the specific contents of the cell are probably either increased in quantity, or brought to greater concentration; a stage, in one word, of ripening;—Fifthly, the falling of the cell with its contained material, in the form of excretion." (p. 70.)

It seems that, in ordinary glands, the cell-membrane is less completely formed than it is in most other instances; so that it more easily liquefies and sets free its contents; and in certain cases it seems altogether wanting, the nucleus, with the material developed around it, constituting the sole physical evidence of activity in the part. Hence *this* must be regarded (according to Mr. Simon) as the characteristic and essential part of the apparatus; and the presence of the cell-membrane must be considered as indicating that a certain degree of completeness has been attained. "The act of secretion, though essentially homologous with ordinary molecular nutrition, is peculiarly prone, in various cases and for various reasons, to exhibit its process of cell-growth, in a low and (as it were) aborted form." In the regular glands, the formation of the complete cell is the usual type of secretory nutrition; and though the exceptions are not unfrequent, they can be accounted for by the circumstances of local excitement or of general ill-supply, under which they occur. In the glands without ducts, on the other hand, the development of a perfect cell is exceptional. The following is Mr. Simon's summary of the characters presented by the process, in the four organs of this kind known to physiologists.

"In the malpighian glandules of the spleen,—where the secretion is fluid, and can but rarely be detected by the microscope in a molecular form, where the nutrition fluctuates from hour to hour, and where the assimilative affinities are therefore exerted with equal intensity only for the shortest periods,—a cell rarely, if ever, exists.

"In the thymus,—where also during infancy the secretion is fluid, and where the assimilative acts probably vary in intensity over short and frequent periods, the persistence of cytoblasts without cells seems at first sight equally regular. And such is actually the case during the time of the gland's most active function; but when it becomes comparatively quiescent, or when (as in many reptiles and some mammalia) it assumes the characters of a permanent organ, it will be found that its cytoblasts have undergone their complete development, and become nuclei of the fat-cells which are formed within the limitary membrane.

"In the tubes of the supra-renal glands where the product is solid, there is constant opportunity of observing that transitional stage, in which the secreted matters are closely aggregated in a molecular form around the several cytoblasts; here, too, the completion of a cell is frequent.

"In the vesicles of the thyroid gland, owing to the fluidity of the secretion, no intermediate stage of cell-growth can be seen; but cells, taking the characteristic cytoblasts of the organ for their nuclei, are often developed, and may be seen to contain a fluid of the same nature as that wherein they float." (p. 84.)

Every one must perceive the interest which attaches to this comparison; and must feel that physiologists are indebted to Mr. Simon for a new method for reviewing these curious and perplexing bodies. Whether that method is the correct one, or whether it will have to undergo modification, as the inquiry is extended and made still more precise, it would be premature in us to offer any decided opinion; and we shall content ourselves with exhorting those who have facilities for the investigation, to follow up the same line of inquiry, in regard to the thyroid gland, the spleen, and the supra-renal capsules, which Mr. Simon has prosecuted so successfully with regard to the thymus.

The physiological conclusions arrived at by Mr. Simon, and contained in his sixth chapter, strike us as the least satisfactory part of his whole essay; yet it is no discredit to him to fail in effecting the complete solution of a problem, which has baffled so many of the greatest men in our profession. In justice to our author, we shall place before our readers a summary of his conclusions, and of the arguments on which they are based; and shall content ourselves with pointing out one or two difficulties which it does not explain, and offering one or two suggestions that may give a different direction to speculation. This we can do with a perfectly clear conscience; as we have no pet theory of our own to support, and shall only be too happy to be furnished with an hypothesis on the subject, that will fit *all* the facts.

Mr. Simon first draws a distinction, which is *not* without a real difference, between the "function" and the "use" of an organ. He defines the "function" as meaning that only which an organ does, viewed absolutely and alone; whilst "use" denotes this action viewed in its relations to the entire system. Thus the function of the stomach (that which it does) is to secrete a certain acid mucus; its use (the application of its function) is to reduce the food to a condition, in which its nutritive principles may be absorbed. The function (thus defined) of the thymus may be stated in these words:

"By means of an apparatus strictly analogous to that of true glands, it secretes into a closed cavity certain particular elements of nutrition. Further the secretion has been shown to occur differently under different circumstances; viz. (1) In most animals it occurs only temporarily. The secreted matter then presents itself in a *fluid form*, and is related to the universal material of nourishment, the liquor sanguinis, by the closest affinity of ultimate chemical composition. (2) In some animals, after discharging the temporary function, the gland gradually passes into the permanent exercise of a different, but analogous, act of assimilation, and manifests its secretion in the *solid form of fat*." (p. 86.)

In both these cases, the *function* is essentially the same, and consists in the secretion of nutrient material; the next question, the one which no examination of the gland itself can determine, but one in replying to which we must be rather guided by the general principles of physiological science, relates to the *use* of this sequestration, and the purpose which the matter thus prepared is to answer in the system. In considering this question, Mr. Simon appears to us to fall into a very grave error at the very outset; in regarding the condition of a young and growing animal as having any parallelism with that of a hybernating animal; and more especially in supposing that there is a relation between them, in the reduction of the waste of the animal tissue to a minimum. Everything seems to us to indicate, that this waste is proportionably *greater* in the young animal than in the adult. The functions of nutrition are all performed with greater activity; the quantity of food assimilated is far larger than is required merely for the increase of the fabric; the molecular interchange is well known to be far more rapid, as is shown especially in the phenomena attending the reparation of severe injuries; and the quantity of effete matter set free in the form of urea (which is, *cæteris paribus*, by far the best measure of the waste of the proteine-tissues) is very much greater during infancy and childhood, than it is in the adult, proportionably to the weight of the body at these periods respectively. With these well-known facts before him, we are surprised that Mr. Simon could have based his speculations, in regard to the function of the thymus, on so insecure a foundation.

Reasoning upon the supposed analogy in the condition of the young, and of the hybernating animal, Mr. Simon arrives at the conclusion that the thymus gland fulfils, in the first case as in the second, the office of a reservoir or sinking-fund of nutritious matter, for the supply of the respiration; and this idea does not seem to him to be inconsistent with the fact, that the product of its separative action is a proteine-compound in the one case, and fatty matter in the other. For he considers that, in consequence of the small amount of *waste* in the tissues of young animals, the support of the respiratory process must chiefly depend upon the materials *directly* supplied by the ingesta; and that as the fatty deposit in hybernating animals constitutes a storehouse of combustible materials, for the prolonged maintenance of the respiratory process, so will the thymus in the young animal keep the balance, amid the hourly changes in the relative amounts of the ingesta, the waste in the tissues, and the respiration, by separating respirable matter from the blood when it is superfluous, and yielding it up again when there is a demand for it.

Having pointed out what we deem the erroneous foundation of Mr. Simon's hypothesis, we shall not stop to discuss the hypothesis itself; but need only remark that the balance in question must be required in the adult, nearly as much as in the young animal; since all the variations of which he speaks are as well marked in the former as in the latter. Let us now endeavour to give to inquiry a more satisfactory direction. In the first place, it must be freely admitted, that neither the body in question, nor any other of the glands without ducts, can have for their office the separation of any elements from the blood, which are destined for immediate excretion; since no channel exists for the deportation of the products of their action, except the sanguiferous system itself. The old idea that the lymphatics served as the excretory ducts of these glands, seems to be quite inconsistent with modern anatomical researches; in regard to the thymus at any rate. Whatever be the action of this gland, therefore, its product must be one that can be readmitted into the current of the circulation without injury. Secondly, the entirely different composition of its products, under the two conditions of its greatest development, seems to indicate that their "use" in the system is not the same. The conditions of young rapidly-growing animals, and that of hybernating animals, seem to us rather *opposite* than *parallel.* The former ingest a large quantity of crude aliment; the assimilating processes are extremely active; and the interchange of the ingredients of the tissues takes place so rapidly, that we cannot imagine that there can be any want of effete matter for respiration. *The demand here is for plastic materials.* On the other hand, in the hybernating animals, all the nutritive actions are at zero; and the respiration, for a long period, is entirely dependent upon the stores of fatty matter which has been previously set apart from the food. *The demand here is for combustible material.* Now the nature of the contents of the thymus, at the two periods, corresponds so precisely with the chief demand which we have shown to exist in the system, that the hypothesis of its being destined to supply these demands appears to us more plausible than the one proposed by Mr. Simon. We have compared it with his under all the circumstances to which he alludes; and we find it at least equally applicable. Take for instance the fact mentioned by Mr. Gulliver, "that in over-driven lambs the thymus will soon shrink remarkably, and be nearly drained of its contents, but will become as quickly distended again during rest and plentiful nourishment." And if the view, to which we have on several former occasions alluded, as to the influence of cell-life upon the production of fibrine from albumen, or, in other words, of the plastic from the non-plastic material of nutrition,—be founded in truth, we have in the thymus gland an organ for carrying on this function during the period when the greatest demand for plastic matter exists. As this demand becomes less energetic, the thymus diminishes in size and disappears,—the production of plastic matter within the absorbent and sanguiferous vessels being then sufficient for the wants of the system. Or if the organ remains, the nature of its "function" changes; and it cannot be deemed unreasonable to suppose that its "use" in the system should change also. In fact, that its "use" should be the same in the two cases, when its "functions" is so different, appears to us a very improbable supposition.

We cannot conclude without again thanking Mr. Simon for the very important contribution which he has made to that *real* knowledge of anatomy, upon which so much of physiological science necessarily rests; nor without saying, to our younger readers especially, "go and do thou likewise." The success which has attended this and other similar investigations, we regard but as an earnest of that which must result from the employment of similar methods in the examination of other parts. The time when a description of an organ with the naked eye, or with an ordinary magnifier, could be satisfactory to the anatomist, has long since past; and no monograph like the present can be in future accepted as complete, in which the microscopical and chemical analysis of the organ is not united with an investigation of its comparative structure in all the leading forms of animals that possess it.

